# Leukaemia inhibitory factor is associated with treatment resistance in oesophageal adenocarcinoma

**DOI:** 10.18632/oncotarget.25950

**Published:** 2018-09-14

**Authors:** Amy M. Buckley, Niamh Lynam-Lennon, Susan A. Kennedy, Margaret R. Dunne, John J. Aird, Emma K. Foley, Niamh Clarke, Narayanasamy Ravi, Dermot O’Toole, John V. Reynolds, Breandán N. Kennedy, Jacintha O’Sullivan

**Affiliations:** ^1^ Department of Surgery, Trinity Translational Medicine Institute, Trinity College Dublin, Dublin, Ireland; ^2^ Department of Histopathology and Morbid Anatomy, School of Medicine, Trinity College Dublin, Dublin, Ireland; ^3^ Department of Clinical Medicine, Trinity Translational Medicine Institute, Trinity College Dublin, Dublin, Ireland; ^4^ UCD Conway Institute & UCD School of Biomolecular and Biomedical Science, University College Dublin, Dublin, Ireland

**Keywords:** LIF, oesophageal cancer, LIFR, treatment resistance, radiation

## Abstract

Oesophageal cancer is an aggressive disease with a poor 5 year survival rate of <20% of diagnosed patients. Unfortunately, only 20-30% Oesophageal Adenocarinoma (OAC) patients show a beneficial response to neoadjuvant therapy (neoCT). Inflammation influences OAC given the increased risk of cancer development and poor outcome for obese patients where altered secretion of adipokines and cytokines from adipose tissue contributes a pro-tumourigenic environment. We carried out a large proteomics screen of 184 proteins to compare the inflammatory and oncogenic profiles of an isogenic radioresistant *in-vitro* model of OAC. We found that leukaemia inhibitory factor (LIF), an IL-6 type cytokine, was significantly elevated in radioresistant OAC cells (p=0.007). Furthermore, significantly higher circulating levels of LIF were present in the serum from treatment-naive OAC patients who had a subsequent poor pathological response to neo-adjuvant therapy, (p=0.037). Quantitative PCR analysis revealed expression of LIF receptor (LIFR) may function as a predictive indicator of response to neo-adjuvant chemoradiation therapy in OAC. LIF was demonstrated to be actively secreted from human OAC treatment-naïve biopsies and significantly correlated with the secretion of bFGF, VEGF-A and IL-8 (p<0.05, R=1), (p<0.05, R=0.9429), and (p<0.05, R=1) respectively. Importantly, LIF secretion negatively correlated with tumour infiltrating lymphocytes in pre-treatment OAC patient biopsies, (r=-0.8783, p=0.033). Elevated circulating LIF is a marker of poor response to neo-adjuvant treatment in OAC and secretion of this chemokine from the tumour is tightly linked with pro-tumourigenic mediators including bFGF, VEGF-A and IL-8. Targeting this pathway may be a novel mechanism enhance neoadjuvant treatment responses in OAC.

## INTRODUCTION

Oesophageal cancer is the 8th most common cancer worldwide with approximately 456,000 new cases diagnosed annually [1]. Oesophageal cancer is an aggressive disease and the 6^th^ most common cause of cancer related death, accounting for approximately 400,000 deaths annually [1]. Oesophageal cancer is classified into two histological subtypes, squamous cell carcinoma (SCC) and oesophageal adenocarcinoma (OAC) [2]. Whilst SCC is the predominant subtype globally in western populations, the incidence of OAC has increased by approximately 48% over the past 15 years [1, 2]. The best outcomes are associated with early disease diagnosis [1, 2].

The current standard of care for OAC focuses on neoadjuvant treatment with chemotherapy (neoCT) alone or in combination with radiation; neoadjuvant chemoradiation (neoCRT) for locally advanced tumours, prior to surgery [3]. The MAGIC chemotherapy protocol consists of the administration of Epirubicin, Cisplatin or Oxaliplatin, and 5-Fluorouracil/or Capecitabine chemotherapy pre- and post-operatively, the CROSS protocol consists of the administration of carboplatin and paclitaxel chemotherapy with fractionated radiotherapy of 41.4 Gy over five weeks [4, 5]. A Cancer Trials Ireland-sponsored randomised, phase III clinical trial, Neo-AEGIS, is comparing neoadjuvant and adjuvant chemotherapy (MAGIC protocol) to neoadjuvant CRT (CROSS protocol) in OAC [6]. Surgery offers the best chance of loco-regional control and neoadjuvant treatment aims to reduce tumour burden prior to surgery to improve post-operative outcome, neoCRT in combination with surgery has been associated with higher rates of overall survival [3, 7, 8]. Unfortunately, only 20-30% of patients show a complete pathological response (pCR) to neo-adjuvant therapy with 70-80% of patients receiving a toxic treatment with little to no therapeutic gain and a subsequent delay to surgery [9–11]. Importantly, there are currently no clinico-pathological markers available to stratify patients who will achieve a beneficial response to radiation therapy.

Ionizing radiation (IR) is a crucial treatment modality used to exert local tumour control in over 50% of human malignancies [9]. IR primarily aims to exert local control through the induction of cellular DNA damage including critical double strand breaks (DSB) [9]. Response to radiation plays a central role in patient outcome in OAC, sensitivity of IR is inversely correlated to tumour burden [12]. Resistance to radiation therapy is polymodal and associated with a number of biological alterations both within the tumour itself and the surrounding microenvironment including; altered cell cycle [13] accelerated repopulation [14, 15], hypoxia [16], evasion of apoptosis [17], altered DNA damage response and enhanced DNA repair [18], and altered mitochondrial function and cellular energetics [19]. In OAC, altered mitochondrial function and DNA repair have been specifically linked to with a radio-resistant phenotype [18, 19]. Furthermore, OAC has been identified as an inflammatory-driven upper gastrointestinal cancer and previous studies have reported the role of inflammation as a negative regulator of response to radiation treatment in OAC [20, 21]. C3a and C4a, components of the complement system, were previously found to be upregulated in the pre-treatment serums of OAC patients having a subsequent poor pathological response to neoCRT, when compared to patients having a good response treatment [21].

A study by Liu *et al.* reported a potential role of the inflammatory cytokine leukaemia inhibitory factor (LIF) pathway in radioresistance of nasopharyngeal cancer (NPC), elevated serum levels were associated with significantly poorer recurrence-free survival [22]. LIF is an IL-6 type cytokine which signals through the leukaemia inhibitory factor receptor (LIFR) and glycoprotein (gp)-130 [23]. Other members of this family include IL-6, IL-11, cardiotrophin-1, cardiotrophin-like cytokine, ciliary neutrophic factor and oncostatin M [23]. Activation of the LIFR pathway is associated with the activation of a number of downstream pathways including the ERK1/2, JAK/STAT3 pathway, MAPK pathway and PI3K/AKT pathway [22, 24, 25]. Differential expression of LIF and/or LIFR is reported in a number of cancers including breast cancer, colorectal cancer (CRC), NPC, osteosarcoma, pancreatic cancer, melanoma, cholangiocarcinoma and cervical cancer [22, 24, 26–31].

LIF is a multifunctional protein and its role is often context-dependent. For example, in non-pathological conditions LIF plays an important role in embryonic implantation where dysregulated LIF expression links to implantation failure [32]. Furthermore in cancer, the role of LIF is complex and linked to both pro- and anti-tumorigenic functions dependent on the cancer type [26, 27, 29]. In breast cancer, LIF can promote tumour growth and migration *in-vitro* and *in-vivo* [24]. In addition, ectopic over-expression of LIF in CRC reduces chemotherapy-induced apoptosis in a p53-dependent manner [27]. In contrast, in cervical cancer, elevated LIF expression is associated with a reduction in cellular proliferation mediated by the downregulation of human papillomavirus-16 (HPV-16) oncogene expression [29]. However the role of LIF in OAC disease progression and treatment response has not yet been explored.

This study aimed to investigate the association of the pro-inflammatory cytokine LIF with response to neo-adjuvant treatment in OAC, in both *in-vitro* settings and in pre-treatment OAC patient serum and biopsies. We profiled the expression and secretion of LIF *in-vitro, in-vivo and ex-vivo*. LIF expression and secretion was upregulated in radioresistant cells of an isogenic model of OAC radioresistance *in-vitro*. *In-vivo*, circulating LIF was significantly elevated in pre-treatment serum from OAC patients with a subsequent poor response to neo-adjuvant treatment. *Ex-vivo*, LIF secretions from treatment naïve biopsies were positively correlated with secretions of IL-8, bFGF and VEGF-A. LIF secretions *ex-vivo* were negatively correlated with percentage lymphocyte infiltration into the tumour biopsies. In addition to LIF, downregulated LIFR expression is significantly associated with poor response to neoCRT in OAC pre-treatment biopsies. Our findings both *in-vitro* and in patient samples strongly implicate the LIF/LIFR pathway in treatment response in OAC which warrants further investigation as a therapeutic target.

## RESULTS

### LIF and LIFR expression is elevated in radioresistant OAC cells

To investigate the role of inflammatory and oncogenic mediators in radioresistance of OAC, we carried out a comprehensive proteomics screen using a previously described isogenic *in-vitro* model of OAC radioresistance [18]. The radioresistant OE33R cells, which were previously chronically irradiated, show significant resistance to radiation when compared to the parental OE33P cells, radiation sensitive cells. This isogenic cell line provides a unique model to investigate cellular and molecular mediators involved in radioresistance in OAC [18].

**Figure 1:**
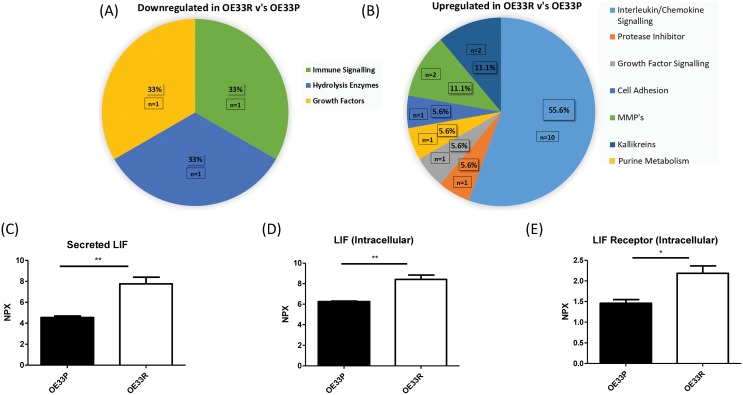
Secreted LIF and intracellular levels of LIF and LIF receptor (LIFR) are significantly higher in OE33R, radioresistant OAC cells compared to OE33P, and radiation sensitive cells **(A)** Pie chart illustrating factors significantly downregulated intracellularly in OE33R (radioresistant) cells versus OE33P (radiation sensitive) cells (p<0.05). **(B)** Pie chart illustrating factors significantly upregulated intracellularly in OE33R cells versus OE33P cells (p<0.05). **(C)** Expression levels of secreted LIF in supernatant from OE33P radiation sensitive and OE33R; radiation resistant OAC cells (n=3) **(D)** Expression levels of LIF intracellularly in OE33P and OE33R cell lysates (n=3). **(E)** Expression levels of the LIFR intracellularly in OE33P and OE33R cell lysates (n=3). Unpaired t-test, ^*^p<0.05, ^**^p<0.01. NPX: Normalised Protein Expression.

Given the multifaceted role of inflammation in cancer progression, we investigated the levels of 184 oncogenic and inflammatory proteins in the supernatants and cell lysates of isogenic OE33P and OE33R cells using a multiplex system. This broad screen of 184 inflammatory and oncogenic proteins found 3 proteins significantly downregulated and 21 proteins significantly upregulated intracellularly in cell lysates in OE33R; radioresistant cells, compared to radiation sensitive OE33P cells (Figure 1A, 1B). Proteins significantly downregulated were linked with immune signalling, hydrolysis and growth signalling (Figure 1A). A greater number of proteins were significantly upregulated in OE33R; radioresistant cells and are involved in different biological processes with the majority of those identified in this specific study linked with interleukin and chemokine signalling (Figure 1B). In particular, the inflammatory profile generated in this screen found that the interleukin 6 type cytokine, LIF, was significantly upregulated in radioresistant OE33R cells in terms of both secretion and intracellular expression (p=0.007, p=0.006), respectively, when compared to OE33P cells (Figure 1C, 1D). In addition, the LIF receptor, LIFR, was significantly upregulated (p=0.022) intracellularly in OE33R cells relative to OE33P cells (Figure 1E). This data indicates that the *in-vitro* expression of LIF is associated with a radioresistant phenotype in OAC.

### Secreted LIF and intracellular LIF and LIFR expression is increased in radioresistant OAC cells

**Figure 2:**
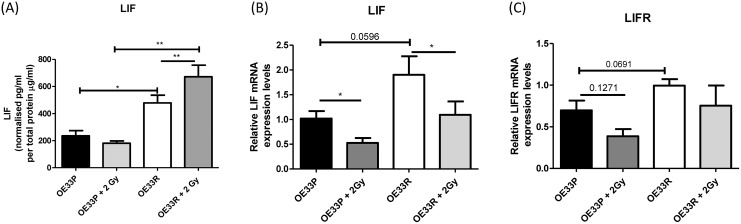
Validation of LIF and LIFR expression data from OLINK screen and investigation of expression and secretion profile of LIF and LIFR at 0 Gy and 24 hours post 2 Gy irradiation **(A)** Secreted LIF protein from OE33P and OE33R cells which have been-mock irradiated and following 2 Gy X-ray irradiation was evaluated by ELISA, (n=4). **(B)** Relative expression of LIF mRNA in OE33P and OE33R cells at baseline and following 2 Gy Irradiation was evaluated by qPCR, (n=5). **(C)** Relative expression of LIFR mRNA at baseline in OE33R and OE33P cells and post 2 Gy irradiation was evaluated by qPCR, (n=5). Unpaired t-test used to compare OE33P and OE33R cell lines, Paired t-test used to compare within same cell lines. ^*^p<0.05, ^**^p<0.01.

We sought to validate the data generated in the multiplex screen and to investigate secretion and intracellular expression profiles of LIF and LIFR post-irradiation. Secreted levels of LIF protein from OE33R cells were significantly higher when compared to OE33P cells at 0 Gy (p=0.012) and 24 hours post 2 Gy X-ray radiation (p=0.001), (Figure 2A). This result validated the screen data and illustrated that radiation significantly increased the secretion of LIF specifically in the radiation resistant OE33R cells (p=0.008) but no significant change was seen following 2 Gy irradiation in radiation sensitive OE33P cells. Given that baseline LIF expression is higher in OE33R cells and that radiation significantly induces LIF secretion in OE33R cells this result indicates LIF is an important molecular mediator of response to radiation in OAC. Supporting the protein data, LIF mRNA expression, as evaluated by RT-PCR, was elevated in OE33R cells compared to OE33P cells (p=0.059) (Figure 2B). Interestingly, 24 hours after the cells were exposed to one dose of 2 Gy irradiation, the levels of LIF mRNA expression significantly decreased in both OE33P (p=0.034) and OE33R cells (p=0.013). Similar to our findings from the proteomics screen, LIFR expression was higher in OE33R cells and no significant change in expression was observed following 2 Gy irradiation (Figure 2C). Our findings suggest that LIF and LIFR may be expressed at higher levels in radiation-resistant OAC cells when compared to radiation sensitive cells although this is not significant, and that radiation treatment significantly increases the secretion of LIF by OE33R cells.

### Significantly increased levels of circulating LIF and decreased levels of tumoural LIFR expression are associated with a poor response to neoadjuvant treatment in OAC

**Figure 3:**
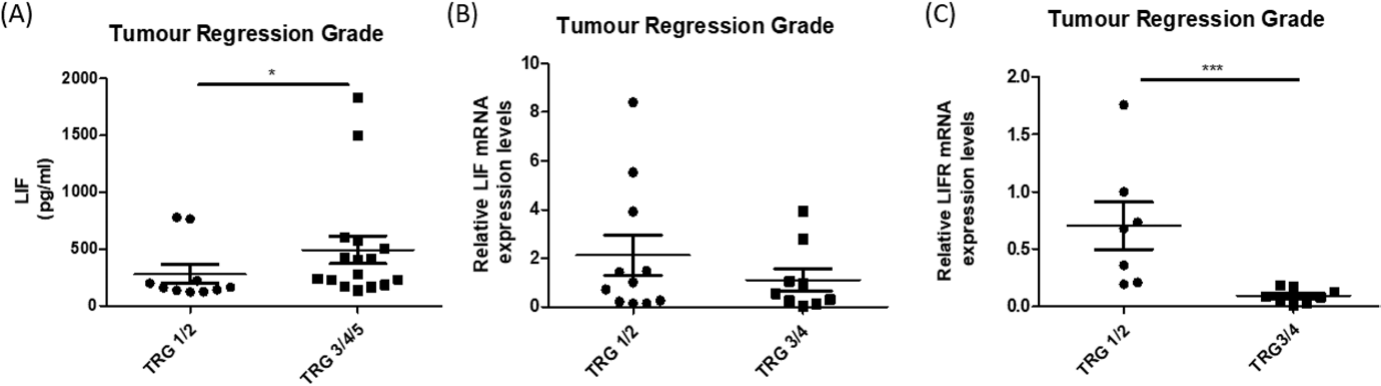
Circulating levels of LIF and tumoural levels of LIFR are significantly altered in OAC patients with a subsequent poor response to neo-adjuvant treatment **(A)** Circulating LIF in pre-treatment serum of OAC patients was assessed by ELISA. Patients were divided into good (TRG 1/2) (n=10) and poor responders (TRG 3/4/5) (n=16) based on pathological response to neo-adjuvant treatment. **(B)** Relative mRNA expression of tumoural LIF in OAC pre-treatment biopsies from good (TRG 1/2) (n=11) and poor (TRG 3/4) (n=9) responders (n=20). **(C)** Relative mRNA expression of tumoural LIFR in OAC pre-treatment biopsies from good (TRG 1/2) (n=6) and poor (TRG 3/4) (n=10) responders. Statistical analysis was performed by Mann-Whitney U test. ^*^p<0.05, ^***^p<0.001.

The levels of circulating LIF in the serum of treatment-naïve patients was evaluated in 26 OAC patient samples by ELISA, the patient cohort is outlined in Supplementary Table 1. Circulating levels of LIF were significantly elevated in patients who went on to have a subsequent poor pathological response following neo-adjuvant treatment (neoCT or neoCRT), with a Mandard tumour regression grade (TRG) of 3-5, compared to patients who had a subsequent good pathological response to neo-adjuvant treatment, with a TRG of 1-2 (p=0.037) (Figure 3A). Circulating levels of LIF were not significantly associated with other patient clinical characteristics, such as tumour stage, nodal status and stage of differentiation (Supplementary Figure 1). In contrast to circulating levels of LIF in serum, there was no significant difference in LIF mRNA expression in pre-treatment OAC tumour biopsies from good and poor responders to neoadjuvant treatment (Figure 3B) indicating that LIF in the circulation may be a more important predictive marker of treatment response than expression levels of LIF within the tumour. Furthermore, LIF expression was not significantly associated with other patient characteristics such as tumour stage, nodal status, stage of differentiation or body mass index (Supplementary Figure 2). LIFR expression (detected in 16 of 24 patients all of whom received neoCRT) was significantly reduced in tumour biopsies from patients having a subsequent poor pathological response to neoCRT (p<0.001), when compared to good responders (Figure 3C). Patient cohort for mRNA expression of LIF and LIFR is outlined in Supplementary Table 2. Circulating LIF and intra-tumoural LIFR expression was associated with neoCRT treatment response but not with other patient clinical characteristics (Supplementary Figures 1 and 3). This suggests that circulating LIF and intra-tumoural LIFR expression may function as valuable pre-treatment predictive indicators of response to neoadjuvant therapy.

LIF secretions were significantly correlated with the levels of secreted basic fibroblast growth factor (bFGF), vascular endothelial growth factor (VEGF-A) and IL-8 in OAC treatment-naïve human tumour explants *ex-vivo*

**Figure 4:**
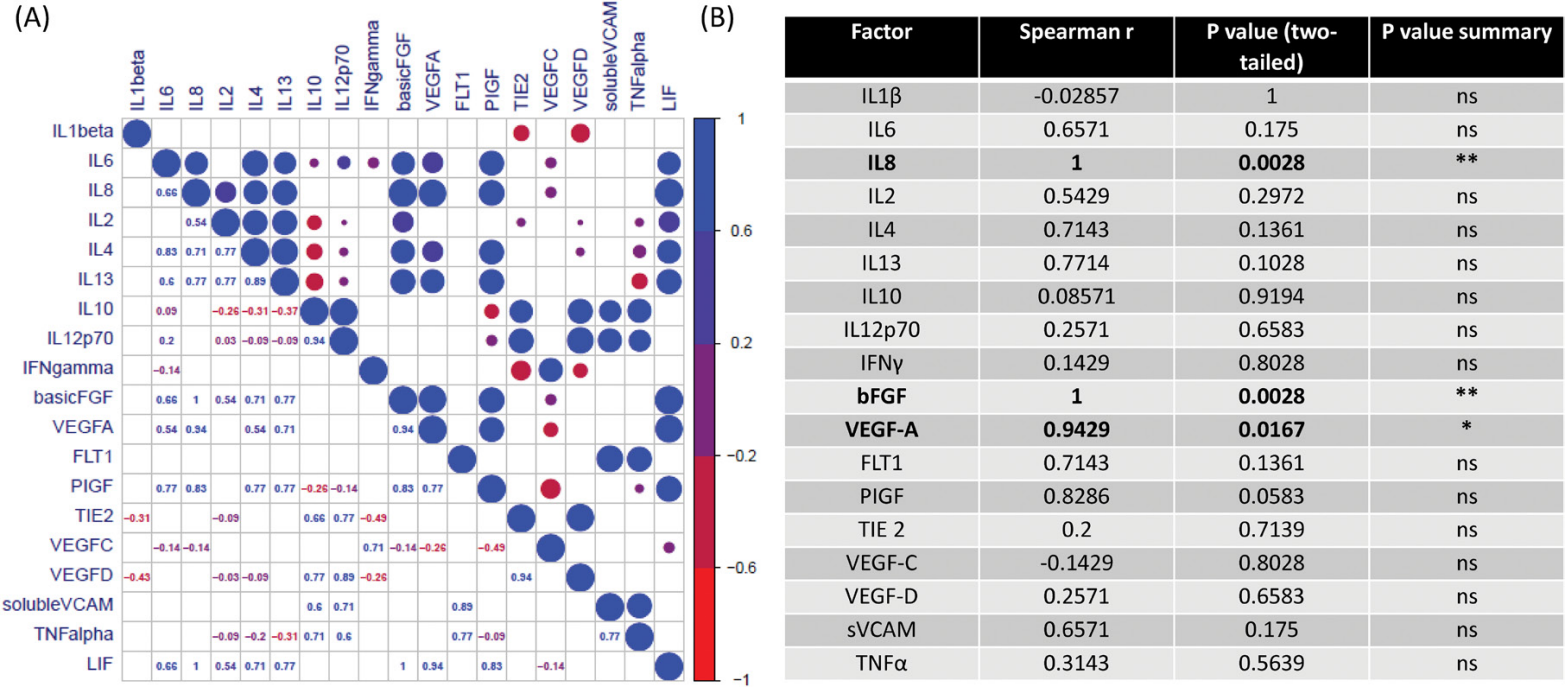
LIF secretions are significantly associated with the secretion bFGF, VEGF-A and IL-8 *ex-vivo* **(A)** CorrPlot illustrating the correlation values of secreted LIF to secreted angiogenic and inflammatory secretions evaluated by ELISA from OAC treatment naïve tumour biopsies cultured *ex-vivo* (n=6). Blue indicates a positive correlation, red negative correlation. **(B)** Table showing Correlation of secreted LIF secreted angiogenic and inflammatory secretions in OAC treatment naïve biopsies cultured *ex-vivo* (n=6). Secretion of inflammatory and angiogenic mediators was determined by ELISA. Spearman correlation to LIF secretions where Spearman r 0.4-0.59 moderate, 0.6-0.79 strong and 0.8-1 very strong. ^*^p<0.05, ^**^p<0.01.

Given the importance of circulating LIF as a predictive marker of treatment response we further sought to investigate secreted levels of LIF from OAC patient tumours using a human *ex-vivo* model using treatment-naïve OAC patient tumour biopsies (patient cohort outlined in Supplementary Table 3). This unique model most closely recapitulates the tumour microenvironment, encompassing multiple cell types as seen *in-vivo*. *Ex-vivo* treatment-naïve human explant tissue was cultured for 6 OAC patients, and the secretions of LIF and a panel of inflammatory (n=10) and angiogenic (n=8) secretions in this Tumour Conditioned Media (TCM) were evaluated by ELISA. The secreted levels of these mediators were correlated in order to ascertain what other mediators in the *ex-vivo* tumour microenvironment were associated with LIF. We demonstrate for the first time that LIF is actively secreted from OAC tumour biopsies (range: 24.61 1137.36 pg/mL) and significantly correlates with the levels of basic fibroblast growth factor (bFGF), VEGF-A and IL-8 (p<0.05, R=1) (p<0.05, R=0.9429) (p<0.05, R=1) respectively, (Figure 4A, 4B). Our studies *ex-vivo* have importantly demonstrated that LIF is secreted into the tumour microenvironment in OAC and is significantly positively correlated with pro-angiogenic and growth factors secretions.

### Elevated secreted LIF negatively correlates with lymphocyte infiltration in human OAC pre-treatment biopsies

**Figure 5:**
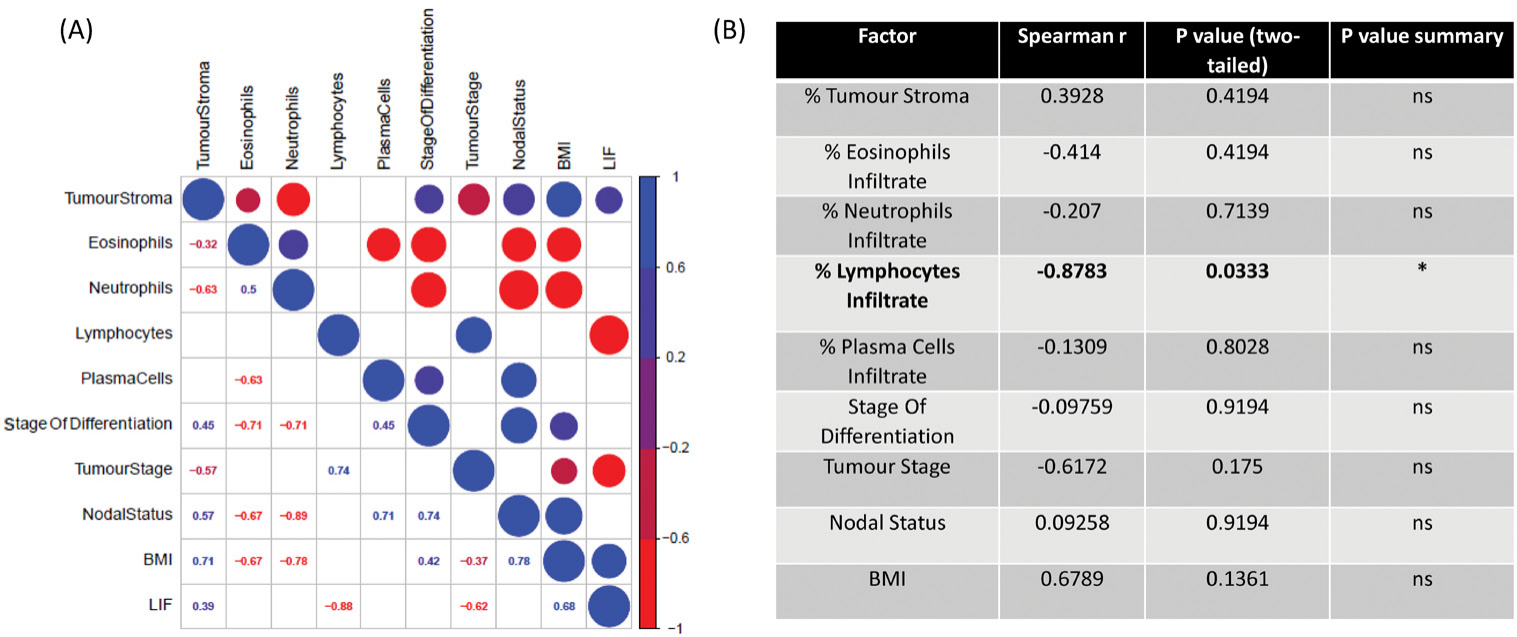
Elevated levels of secreted LIF negatively correlates with lymphocyte infiltration in human OAC pre-treatment biopsies **(A)** CorrPlot illustrating the correlation values of secreted LIF from OAC treatment naïve biopsies cultured *ex-vivo* to patient clinical characteristics (stage of differentiation, tumour stage, nodal status and BMI) and percentage tumour stroma immune cell infiltrations (neutrophils, eosinophils, lymphocytes and plasma cells). Blue indicates a positive correlation, red negative correlation, (n=6) **(B)** Table showing Correlation values of secreted LIF to patient clinical characteristics and immune cell infiltrate (n=6). Spearman correlation to LIF secretions where Spearman r 0.4-0.59 moderate, 0.6-0.79 strong and 0.8-1 very strong.^*^p<0.05.

Following our *ex-vivo* studies using TCM generated from 6 patient OAC pre-treatment biopsies, we sought to investigate the association of secreted LIF with immune cell infiltrate and patient clinical characteristics. It is critical to understand the role of LIF in the tumour microenvironment, its association with other secreted growth factors and cytokines, and how LIF secretion is associated with immune cell infiltration of tumours. Immune cell infiltrates were determined by a pathologist using matched diagnostic H&E slides prepared from pre-treatment biopsies of 6 patients (patient cohort is outlined in Supplementary Table 3). We observed that LIF secretion was significantly negatively correlated with lymphocytic infiltration whereby lower LIF secretion was associated with greater lymphocyte infiltration in matched biopsies (p=0.0333, r=-0.8783) but no significant association was seen with other types of infiltrating cells, e.g. eosinophils, neutrophils, plasma cells (Figure 5A, 5B).

## DISCUSSION

LIF is a multi-functional cytokine which under non-pathological conditions plays an essential role in embryonic implantation, bone formation and neuronal development [32, 33]. In cancer, previous studies have demonstrated the role of LIF as an oncogenic mediator which stimulates cancer growth, proliferation and metastasis, and have associated LIF with both resistance to radiation and chemotherapy treatments [22, 24, 27]. To our knowledge this is the first study to investigate the role of LIF in OAC. OAC is an aggressive disease with a poor prognosis, currently over 70% of patients don't show a beneficial response to neo-adjuvant therapy [11]. A complete pathological response to treatment is associated with increased survival, thus it is critical to gain further insight into the molecular mediators which play a role in treatment response in OAC.

*In-vitro*, both in our screen and validation study, LIF and LIFR expression was elevated in radioresistant OE33R cells relative to radiation sensitive OE33P cells. Overexpression of LIF at the mRNA level has been previously shown in a number of cell lines such as breast cancer where LIF expression was found to be significantly higher in breast cancer cells with greater metastatic capability [24]. *In-vivo*, targeting of the LIF/LIFR pathway through inhibition of LIFR with small interfering RNA was shown to inhibit metastasis in rhabdomyosarcoma xenografts, further highlighting both the role of LIF in tumourigenesis and therapeutic targeting potential of this pathway [34]. Furthermore in NPC, treatment with soluble LIFR, an antagonist of LIF or rapamycin, an mTOR inhibitor of LIF, was found to reduce cell survival and tumour growth following irradiation *in-vitro* and *in-vivo* [22]. Given these findings in the literature in addition to our studies in OAC, the potential of targeting this pathway to enhance radiosensitivity in OAC must be investigated in future studies.

LIF secretion was significantly higher in OE33R cells than OE33P cells, complementing our findings at the gene level. An important finding from this result was the differential secretion of LIF in OE33P and OE33R cells following one dose of 2 Gy radiation, the response to radiation was cell line specific, with elevated levels of secreted LIF identified in radioresistant OE33R cells only. In contrast, at the gene level LIF expression was reduced in both cell lines 24 hours following 2 Gy irradiation, the response to radiation was cell line specific, with elevated levels of secreted LIF identified in radioresistant OE33R cells only, it would be interesting to evaluate the levels of LIF at an earlier time point following irradiation to see if initially LIF expression is elevated in response to irradiation. Higher LIF secretion in the OE33R cells may result in increased activation of the LIF/LIFR pathway in OE33R cells and could be contributing to the radioresistant phenotype. *In-vivo*, LIF was previously shown to significantly enhance radioresistance in a NPC xenograft model whereby the administration of soluble LIF following one dose of 7 Gy irradiation significantly enhanced resistance to radiation and promoted tumour growth [22]. The enhanced tumour growth following LIF administration is supported by other studies in both breast and pancreatic cancer, where LIF administration and ectopic expression was shown to promote tumour growth and tumour progression *in-vitro* [24, 28]. Our findings, along with the current literature, indicate that LIF may play a role in treatment resistance in OAC. Enhanced cell growth and tumour progression as a result of LIF pathway activation has been linked to downstream activation of the JAK/STAT3 and Akt/mTOR pathways. In these studies, mTOR expression has been identified in both OE33P and OE33R cells previously (data not shown) but the actual pathway through which LIF signals in OAC requires further investigation, especially since inhibition of mTOR has previously been shown to enhance radiosensitivity in oesophageal SCC, lung and pancreatic cancer [24, 35, 36].

In OAC patient pre-treatment serum, circulating LIF was found at significantly higher levels in OAC treatment-naïve patients who went on to have a subsequent poor pathological response to neoadjuvant treatment with a TRG of 3-5. This result complements our findings *in-vitro* whereby elevated LIF protein secretion was associated with radiation resistance. This result was similar to that seen by Liu *et al.*, in NPC where elevated levels of circulating LIF in serum were positively associated with a poor response to treatment and subsequent local tumour recurrence [22]. Our study demonstrates that elevated circulating levels of LIF is associated with poor response to neoadjuvant treatment (MAGIC or CROSS) and may contribute to subsequent disease progression in OAC. Circulating LIF may therefore have important value as predictive marker of treatment response and as a therapeutic target to halt tumour progression, in the future screening with a larger number of patients in each arm of the trial is warranted for validation of these preliminary observations. Our findings are supported by a study which showed that elevated IL-6 in serum, a key oncogenic cytokine from the same family was shown to predict a two-fold increased risk of progression to malignancy from Barrett's oesophagus (BO), a chronic inflammatory condition and pre-disposing risk factor of OAC [37].

LIFR mRNA expression was significantly reduced in treatment naïve OAC tumour biopsies from patients having a subsequent poor pathological response to treatment, suggesting that a loss in receptor expression is associated with poor treatment response. This result may seem surprising given that elevated circulating LIF was demonstrated to be associated with a poor response. However, several other studies support this data. LIFR expression has previously been identified in breast cancer, CRC, gastric cancer, liver cancer and pancreatic cancer [28, 30, 38, 39]. In hepatocellular carcinoma (HCC), LIFR was found to negatively regulate metastasis via the PI3K/Akt pathway and downregulated expression of LIFR was an indicator of poor prognosis [40]. A previous study in a large cohort of metastatic breast cancer patients also found loss of LIFR to be associated with poor clinical outcome [30]. LIFR was found to promote membrane localisation factor Scribble which in turn led to the activation of Hippo signalling and phosphorylation and functional inactivation of the transcriptional co-activator Yes-associated protein 1 (YAP1) and subsequent suppression of tumour metastasis [30]. It has been suggested that LIFR may be reduced in tumour tissues as a result of the promoter undergoing hypermethylation [25]. Furthermore, in pancreatic cancer tissue microarrays, downregulated LIFR was significantly associated with Tumour Node Metastasis (TNM) stage and lymph node metastasis, and silencing of LIFR *in-vitro* reduced colony formation and metastatic potential of pancreatic cancer cells [38].

The significantly lower levels of LIFR expression in OAC patients with a poor response to neoCRT highlights the loss of LIFR as a predictive indicator of poor outcome, similar to findings in other cancers. This result did not reflect our findings *in-vitro* however tumour biopsies reflect the *ex-vivo* tumour microenvironment to a greater extent consisting of multiple cell types and thus more efficiently reflect what is going on in a tumour *in-vivo*. LIFR may act as an additive predictive indicator for treatment response in OAC, however this result would need to be validated in a larger patient cohort.

Therapeutic targeting of this receptor in OAC warrants further investigation, particularly given that *in-vivo* in pancreatic cancer xenografts, primary tumour volume and lung metastasis were increased by LIFR silencing and tumour volume and metastasis were reduced and inhibited respectively when LIFR was overexpressed in primary implanted pancreatic cancer cells [38]. These studies support our data, which suggests that decreased LIFR in pre-treatment OAC tumour biopsies is a predictive marker of poor response to neoadjuvant treatment. However, this requires further validation in a larger independent patient cohort.

Given the predictive importance of LIF as a circulating mediator, we then sought to investigate the role of secreted LIF in the tumour microenvironment from human pre-treatment OAC tumour biopsies which we cultured *ex-vivo*. We demonstrated for the first time that LIF is secreted from OAC tumours. The angiogenic and inflammatory factors produced by OAC tumours play an important role in disease progression and response to treatment. Secreted LIF was significantly correlated with the secreted levels of bFGF, VEGF-A and IL-8 in the matched TCM of treatment naïve OAC tumour explants. bFGF, similar to LIF, is a pleiotropic factor which was shown to promote cell growth, angiogenesis and differentiation and to prevent apoptosis in cancer [41]. LIF and bFGF are potent growth factors which have been previously shown to stimulate cancer growth in osteosarcoma [24, 42]. In osteosarcoma cells, bFGF was found to enhance cancer growth through hyper activation of ERK 1/2 [42]. Both alone and in combination, LIF and bFGF were found to significantly enhance cell growth of osteosarcoma cells. Importantly, when they were administered together, this produced an additive effect on tumour growth, highlighting a synergistic interplay between both factors [42]. Anti-bFGF antibodies were shown to enhance radiosensitivity in oesophageal SCC through a reduction in colony formation *in-vitro* [36]. In addition, targeting of bFGF by a peptide has been shown to improve chemo-sensitivity in CRC [43]. These findings in the literature and within our study show that LIF and bFGF together both play important roles in cancer progression and treatment response in many cancers and may influence both OAC cells and host immunity by functioning as part of the active cytokine network. Given the ability of both bFGF and LIF to enhance radiosensitivity in previous studies and the strong correlation between both factors in OAC, the potential of the combined targeting to enhance radiosensitivity in OAC strongly warrants further investigation.

Furthermore, secreted VEGF-A was significantly correlated with LIF in the OAC tumour microenvironment. VEGF-A is a well-known tumourigenic mediator which plays a key role in angiogenesis, a process tightly linked with treatment resistance. Targeting of VEGF-A *in-vivo* was shown to enhance radiation response in a head and neck xenograft model [44]. It is unsurprising that LIF, a potent tumourigenic growth factor is strongly associated with VEGF-A, given the necessity of tumour vasculature to promote tumour growth and survival. Given the significant relationship we have shown between LIF and treatment resistance in OAC, and the known role of VEGF-A in tumourigenesis and treatment response, the potential of targeting both mediators to enhance response should be explored in future.

In addition, LIF was found to significantly correlate with IL-8, a pro-inflammatory cytokine which signals through the CXCR1/2 receptors. This signalling results in PI3K, MAPK and JAK2 pathway activation, and has been found to promote cell proliferation, angiogenesis and metastasis in xenograft models where administration of an anti-IL-8 monoclonal antibody attenuated tumour growth and metastatic potential [45]. IL-8 expression has previously been correlated with LIF expression in other inflammatory diseases such as psoriasis [46]. Furthermore, both IL-8 and VEGF-A have been shown to play an important role in tumour angiogenesis, a key process involved in treatment resistance which, when inhibited with targeted therapy, can enhance radiation response *in-vivo* [44, 45].

Given the pivotal role of LIF, bFGF, VEGF-A and IL-8 in tumourigenesis and their correlated secretion in OAC, the potential of targeting these growth factors and cytokines to improve patient response to neoadjuvant treatment and to inhibit tumour progression in OAC must be investigated in future studies. Whilst targeting all 4 mediators simultaneously may not be clinically feasible, this study offers insight into potential mediators which could be targeted to enhance radiosensitivity and possible compensatory mediators which may be upregulated in response to such targeting, possibly providing novel mechanistic insight to resistance to mechanisms that may arise following targeting of one of the mediators in isolation. This study also highlights the potential of sequential targeting of these factors to overcome resistance and to improve treatment response.

OAC tumours do not function in isolation and interaction of the tumour with the host plays a significant role in tumour progression. The recent successes of immunotherapies in the clinic highlight the key role of the host immune system in tumour control, thus we sought to investigate the relationship between LIF and immune cell tumour infiltration. LIF secretion from the tumour microenvironment was negatively correlated to percentage of lymphocyte infiltrate in matched OAC pre-treatment biopsies. The negative correlation between LIF and lymphocyte infiltration, where LIF secretion from the tumour microenvironment is increased in patients with low tumour infiltrating lymphocytes (TILs) is a significant finding, given that high lymphocyte infiltration has previously been associated with improved patient outcome in SCC and many other cancer types TILs are thought to play a pivotal role in tumour control through activation of the host anti-tumour immune response [47]. In addition, higher TILs have been associated with improved prognosis in breast, colon, ovarian and lung cancer [48–50]. The relationship between LIF secretion and immune activation is relatively under-explored in cancer, and it is unknown whether elevated LIF secretion in biopsies with reduced lymphocyte infiltrate is a causal relationship or if this is just an association which could have the potential to determine response of patients to treatment [47, 51]. It is however important to note that immune cell infiltration analysis was carried out on pre-treatment diagnostic OAC biopsies which only represent a very small portion of tumour and the invasive edge was not represented in these biopsies and thus inflammatory infiltrate of the invasive edge could not be analysed. It will be critical to evaluate the effect of targeting the LIF pathway, not only in terms of treatment response but also on immune cell activation and infiltration in OAC.

In this study we have shown an association between expression of the IL-6 family member LIF and treatment response in OAC, both in *in-vitro* cell line models, and in pre-treatment OAC patient serum and biopsies. LIF secretion and expression is elevated in radioresistant cells *in-vitro*. Importantly, circulating LIF is elevated in patients with a subsequent poor response to neo-adjuvant treatment, implicating this pathway in treatment resistance in OAC. LIF secreted *ex-vivo*, from human OAC treatment-naïve biopsies, correlates with the secretion of key tumourigenic growth factors, including bFGF, VEGF-A and IL-8, indicating that these factors are tightly associated with one another in the tumour microenvironment of OAC. A significant finding of this study is the negative correlation of LIF, which we have identified as a poor prognostic in OAC, with percentage of tumour infiltrating lymphocytes which have been reported to play a key role in the host response to tumour and serve as a marker of prognosis in multiple cancer types [51]. The use of LIF as a circulating marker of treatment response in OAC needs to be validated in a larger patient cohort following this pilot study. Furthermore, given the increased secretion of LIF from our radioresistant cells following irradiation, and the tight association of secreted LIF with other pro-tumourigenic factors from our OAC patient tumours, targeting of the LIF pathway to boost treatment response warrants investigation in future studies in OAC.

## MATERIALS AND METHODS

### Generation of the OE33P and OE33R cell lines

The human OE33 oesophageal adenocarcinoma cell line was obtained from the European Collection of Authenticated Cell Cultures. The isogenic model of radioresistant OAC; OE33P (radiosensitive) and OE33R (radioresistant) cells was generated, characterised and cultured in our department as previously described [18].

### Preparation of OE33P and OE33R cell lysates for OLINK proseek proteomics analysis

OE33P and OE33R cells were seeded at a density of 2.5 x10^5^ in 6 well plates in 1.5 mL complete Roswell Park Memorial Institute medium (RPMI) (supplemented with 10% FBS and 1% penstrep) at 37°C in 5% CO_2_. At 48 h supernatant was removed and stored at -20°C. Cells were washed twice with 300 μL of ice cold PBS. NP40 cell lysis buffer (Invitrogen) was supplemented on day of use with 100 μL Phenylmethylsulfonyl fluoride (PMSF) (Sigma-Aldrich) per 10 mL cell lysis buffer and 1000 μL protease inhibitor (Sigma-Aldrich) per 10 mL cell lysis buffer. Supplemented ice cold NP40 cell lysis buffer (200 uL) was added to each well and left on ice for 20 min. Cell lysate solutions were pipetted up and down following incubation to aid lysis process. Lysis buffer solute was centrifuged for 20 min at 13,400 x g at 4°C. Lysates was aspirated off and stored at -80°C. 20 μL of each supernatant and cell lysate of equal protein concentration was determined using the Pierce Bicinchoninic acid (BCA) assay and was placed in a MicroAmp plate (Applied Biosystems) and sealed and shipped on dry ice to OLINK proteomics (Uppsala, Sweden).

### Proseek proteomics assay

OLINK proteomics conducted a proseek proteomics screen of our samples, including supernatants and cell lysates from 3 independent experiments obtained from OE33P and OE33R cells. Samples were run on both their Inflammatory I and oncology II platform, with samples screened for expression of a total 184 proteins (92 per panel). All samples were of equal protein concentration prior to shipping to OLINK. The readout of normalised protein expressions values (NPX) was obtained from OLINK following the assay for statistical analysis. The NPX value indicates the relative quantification for that protein and thus can only be used for comparison between samples for each protein and cannot be used for comparing one protein to another. A hit was determined as significant when p<0.05, p values were adjusted for multiple testing using the false discovery rate method.

### Irradiation

Irradiation was performed using a Gulmay Medical X-ray generator, (RS225) (Gulmay Medical), at a dose rate of 3.25 Gray (Gy) per min.

### Patient samples

Following ethical approval (Joint St James's Hospital/AMNCH ethical review board) and written informed consent, diagnostic biopsy specimens were taken from patients with a diagnosis of operable OAC, by a qualified endoscopist prior to neoadjuvant therapy. Histologic confirmation of tumour tissue in biopsies was performed by a pathologist using routine hematoxylin and eosin staining. Patients with a subsequent TRG score received a complete course of neo-adjuvant therapy. All patient samples in the qPCR study received neoCRT according to the CROSS regimen [6]. In the serum study, 16 patients received neoCRT according to the CROSS regimen and 10 patients received neoadjuvant chemotherapy only according to the MAGIC regimen [6]. All patient tumour and serum samples used in this study were taken prior to initiation pre-treatment.

### RNA isolation from OE33P and OE33R cells

RNA was isolated from cell lines using the TRI Reagent^®^, as per the manufacturer's instructions. Cells were seeded at a density of 2.5 × 10^5^ cells/well in 6-well plates and allowed to adhere overnight. 24 h later OE33P and OE33R were either mock irradiated or irradiated with 2 Gy irradiation. RNA was isolated from cells at 24 h post irradiation using TRI Reagent^®^ (Molecular Research Centre, Montgomery Road, OH, USA), as per the manufacturers instructions. The RNA pellet was re-suspended in 30 μL RNAase free molecular grade H_2_0 and stored at -80°C.

### RNA isolation from OAC pre-treatment biopsies

RNA from patient tumour tissue samples was isolated using a miRNeasy RNA isolation kit (Qiagen), as per the manufacturer's instructions. Total RNA was quantified spectrophotometrically using a Nanodrop 1000 spectrophotometer v3.3 (Thermo Fisher Scientific). Total RNA (1.5 μg) was reverse transcribed to cDNA using a High Capacity cDNA RT Kit (Thermo Fisher Scientific).

### RNA quantification of cell lines and OAC patient mRNA

RNA quantification was determined spectrophotometrically, using a Nanodrop 1000 spectrophotometer (version 3.1.0, Nanodrop technologies, DE, USA). 1 μL RNase-free water was used to blank the instrument prior to RNA analysis. 1 μL of each sample of isolated RNA was loaded onto the instrument and concentration was measured in ng/μL. 260:280 and 260/230 ratios were also recorded to evaluate RNA quality.

### cDNA synthesis from cell lines and OAC patient mRNA

For cell line samples, total RNA (1 μg total RNA) was reverse transcribed to cDNA using the following method. To anneal the primers to the RNA, the sample was heated for 10 min at 70°C, and immediately chilled for at least 1 min at 4°C. A master mix containing RNaseOUT (Invitrogen, Carlsbad, CA, USA) recombinant ribonuclease inhibitor (1unit/μl), dNTPs (Invitrogen, Carlsbad, CA, USA) (10 mM, prepared as a 1:1:1:1 ratio of dATP, dGTP, dTTP and dCTP), Bioscript reverse transcriptase (200units/μl) (Bioline, Kilkenny, Ireland) and 5X Bioscript Reaction Buffer (Bioline, Kilkenny, Ireland) in RNase-free water was added to each sample. Samples were incubated for 1 h at 37°C then 10 min at 70°C and held at 4°C. The resulting cDNA was stored at -20°C. For patient samples, total RNA (1.5 μg) was reverse transcribed to cDNA using a High Capacity cDNA RT Kit (Thermo Fisher Scientific).

### Quantitative real time PCR of cell lines and OAC patient samples

qPCR was performed using TaqMan primer probes and a Quant Studio 5 real-time thermal cycler (Thermo Fisher Scientific). 18S was used as an endogenous control for data normalization. PCR data were analyzed by the 2^–ΔΔCt^ Livak method [52].

### Generation of OE33P and OE33R cell supernatants for ELISA

Cells were seeded at a density of 2.5 × 10^5^ cells/well in 6-well plates and allowed to adhere overnight at 37°C in 5% CO_2_. After 24 hours, OE33P and OE33R were either mock irradiated or irradiated with 2 Gy irradiation. 24 h later supernatant was removed and stored at -20°C. Cells were washed twice with 300 μL of ice cold PBS. RIPA cell lysis buffer was supplemented on day of use with 100 μL phenylmethylsulfonyl fluoride (PMSF) (Sigma-Aldrich) per 10 mL cell lysis buffer and one protease inhibitor cocktail tablet (Roche) per 10 mL cell lysis buffer. 200 μL of supplemented ice cold RIPA cell lysis buffer was added to each well and left on ice for 20 min. Cell solutions were pipetted up and down following incubation to aid cell lysis. Lysis buffer solute was incubated for 20 min at 13,400 x g at 4°C. Lysates were aspirated and stored at -80°C. Protein concentration was determined by BCA assay (Pierce).

### Serum sampling

Following informed, written consent, OAC patient treatment naïve serum was collected using Z-clot activator serum tubes (Greiner Bio-One, Gloustershire, United Kingdom). Samples were centrifuged at 1150 ×g for 10 min, the serum decanted, aliquoted, and subsequently stored at -80°C in a designated biobank.

### Generation of tumour conditioned media

Following informed consent, biopsies were collected at endoscopy, immediately placed on saline-soaked gauze and were transported within 10 minutes to the laboratory. Biopsies were placed in culture as follows: biopsies were placed into a well of a 12 well plate containing 1 mL M199 medium (Gibco) supplemented with 10% FBS (Gibco), 1 μg/mL insulin and 1% penicillin/streptomycin. Following 24 hour culture, Tumour conditioned media (TCM) was collected stored at -80°C. The remaining tissue was snap-frozen in liquid nitrogen and stored at -80°C.

### Enzyme linked immunosorbent assay (ELISA)

Supernatant from OE33R and OE33P cells, OAC patient treatment naïve TCM and OAC treatment naïve serums were defrosted on ice. The secretion of cytokines and angiogenic growth factors was analysed by ELISA as per the manufacturer's instructions. To assess the secretion of LIF from OE33P and OE33R cell supernatants, patient serum and TCM, an individual LIF ELISA kit was used (LifeSpan Biosciences). To assess angiogenic and inflammatory cytokine release, 2 multiplex kits were used (Meso Scale Diagnostics, USA). For angiogenic markers, a 7-plex ELISA was used to quantify levels of bFGF (basic), Flt-1/VEGFR-1, PlGF, Tie-2, VEGF-A, VEGF-C, VEGF-D. For inflammatory cytokines, a 10-plex assay was used to quantify IFN-γ, IL-10, IL-12p70, IL-13, IL-1β, IL-2, IL-4, IL-6, IL-8 and TNF-α. ICAM secretions were detected by a separate ELISA (R&D Systems). Secretion data for all factors was normalised appropriately to cell lysate protein content and explant protein content using the BCA assay (Pierce) for cell supernatant and TCM secretions, respectively.

### Evaluation of inflammatory infiltrate in OAC pre-treatment biopsies

Routine haematoxylin and eosin stained sections from diagnostic biopsy material were reviewed by a pathologist who was blinded to clinical outcomes. Inflammatory cell density and tumour stroma percentage were assessed in tissue fragments containing invasive carcinoma. The inflammatory cell density was classified as either absent, low-grade (mild/patchy increase in inflammatory cells) or high-grade (prominent inflammatory infiltrate and/or involvement and destruction of cancer cell islands). The presence of eosinophils, neutrophils, lymphocytes and plasma cells was also assessed and similarly classified as either absent, low-grade or high-grade. The tumour stroma percentage (TSP) was assessed by estimating the proportion of stroma as a percentage of the visible field from an area of carcinoma, excluding areas of mucin deposition or necrosis. Tumours were classified as low-TSP if stroma accounted for 50% of the visible field or high-TSP if stroma accounted for 50% of the visible field.

### Statistical analysis

Statistical analysis was performed using GraphPad Prism version 5 software (GraphPad Software, CA, USA). Scientific data were expressed as mean ± standard error of the mean (SEM). SEM was calculated as the standard deviation of the original samples divided by the square root of the sample size. Specific statistical tests used are indicated in figure legends. Correlations were carried out using R software version 3.4.1. Graphical representations of correlations were generated with the R package ‘corrplot’. For all statistical analysis, differences were considered to be statistically significant at p<0.05.

## SUPPLEMENTARY MATERIALS FIGURES AND TABLES



## Abbreviations

bFGF: Basic Fibroblast Growth Factor
CRC: Colorectal Cancer
IL-8: Interleukin 8
LIF: Leukemia Inhibitory Factor
LIFR: Leukemia Inhibitory Factor Receptor
HCC: Hepatoceullar Carcinoma
ICAM: Intracellular Adhesion Molecule 1
mTOR: Mammalian Target of Rapamycin
OAC: Oesophageal Adenocarcinoma
NeoCRT: Neoadjuvant Chemoradiation Therapy
NPC: Nasopharyngeal Cancer
SCC: Squamous Cell Carcinoma
TRG: Tumour Regression Grade
TCM: Tumour Conditioned Media
VEGF: Vascular Endothelial Growth Factor.
