# Supplementary material for: Leukaemia inhibitory factor is associated with treatment resistance in oesophageal adenocarcinoma
Source: Oncotarget. 2018 Sep 14;9(72):33634–47. doi: 10.18632/oncotarget.25950 (PMC6154751; doi:10.18632/oncotarget.25950)
Supplement: Supplementary file 1 [file oncotarget-09-33634-s001.pdf]

Leukaemia inhibitory factor is associated with treatment resistance in oesophageal adenocarcinoma

SUPPLEMENTARY MATERIALS

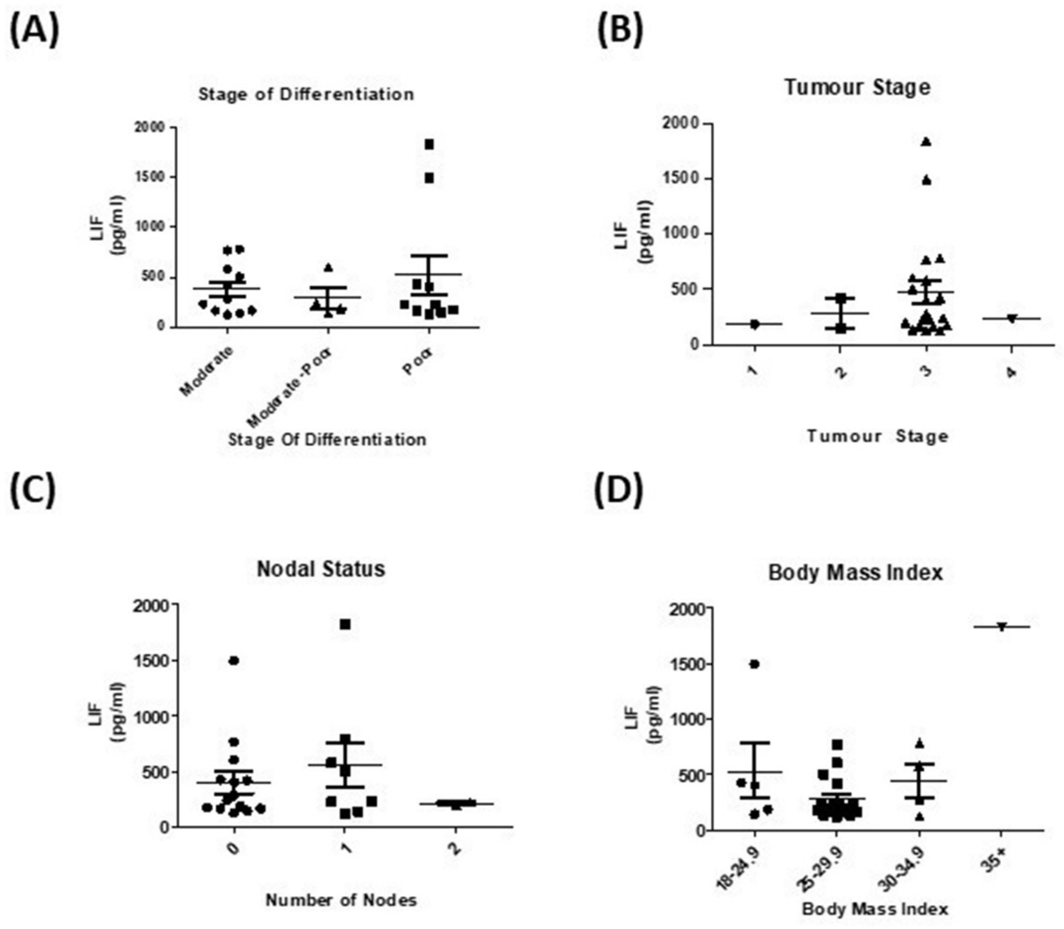

Supplementary Figure 1: Circulating LIF in serum from treatment-naïve oesophageal adenocarcinoma patients, divided according to pathological parameters. Circulating LIF in serum was assessed by ELISA and divided according to **(A)** Stage of Differentiation (n=25), **(B)** Tumour stage (n=26), **(C)** Nodal Status (n=26) and **(D)** Body Mass Index (n=26). Mann-Whitney U test. Data are expressed as mean ± SEM.

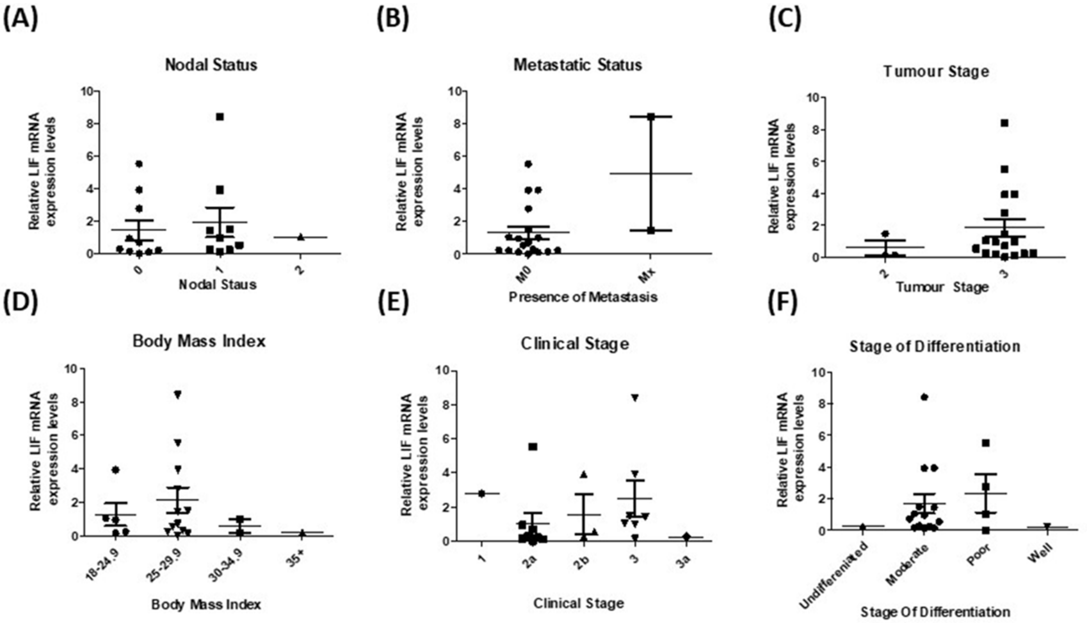

Supplementary Figure 2: Relative expression of LIF in OAC pre-treatment biopsies. Relative expression of LIF mRNA in patient tumour biopsies was divided according to clinical characteristics **(A)** Tumour Stage (n=20) **(B)** Nodal Status (n=20) **(C)** Presence of Metastasis (n=20) **(D)** Body Mass Index (n=20) **(E)** Clinical Stage (n=20) **(F)** Stage of Differentiation (n=20). Mann Whitney U test. Data expressed as mean ± SEM.

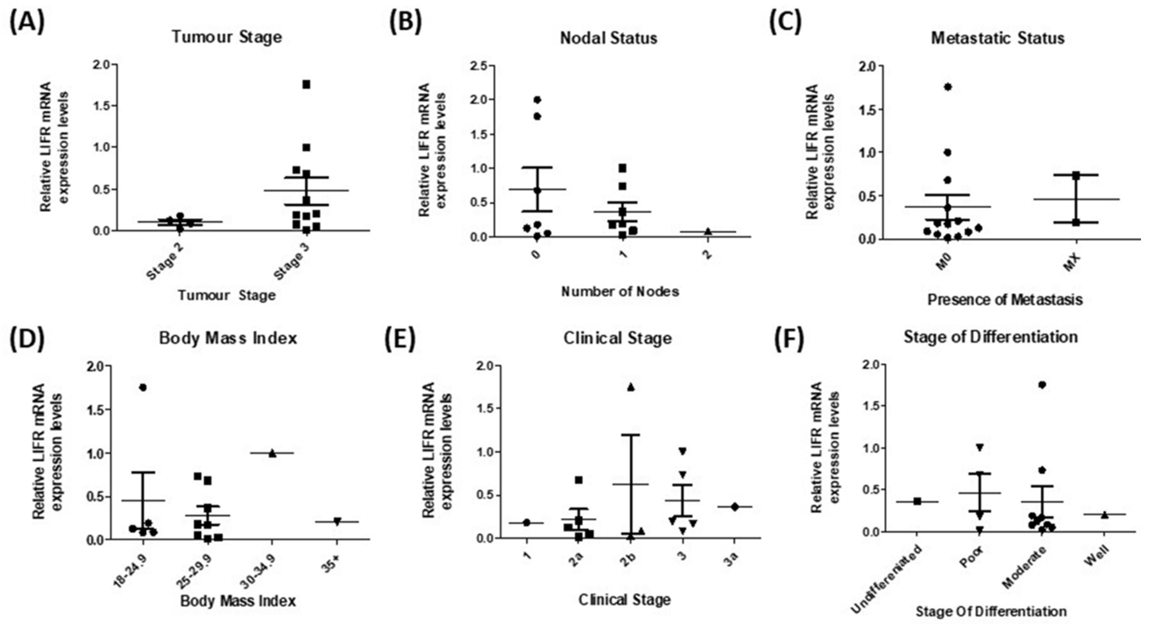

Supplementary Figure 3: Relative expression of LIFR in OAC pre-treatment biopsies. Expression of LIF mRNA in patient tumour biopsies was divided according to clinical characteristics **(A)** Tumour Stage (n=15) **(B)** Nodal Status (n=15) **(C)** Presence of Metastasis (n=15) **(D)** Body Mass Index (n=15) **(E)** Clinical Stage (n=15) and **(F)** Stage of differentiation (n=15).Mann-Whitney U test. Data expressed as mean ± SEM.

Supplementary Table 1: Patient characteristics of patient cohort for RT-PCR study

	**n=24**
Male	20
Female	4
Median Age at diagnosis	62.5 (43-75)
Median BMI at diagnosis	27.34 (20.04-36.61)
Tumour Stage at diagnosis	
2	3
3	21
Nodal Status at diagnosis	
0	12
1	11
2	1
Treatment	
CROSS	24
Tumour Regression Grade	
1	4
2	8
3	6
4	6

Supplementary Table 2: Patient characteristics of patient cohort for serum study

	**n=26**
Male	23
Female	3
Median Age at diagnosis	64.5 (58-80)
Median BMI at diagnosis	27 (23-36.8)
Tumour Stage at diagnosis	
1	1
2	2
3	20
4	1
Not Specified	2
Nodal Status at diagnosis	
0	14
1	8
2	2
No Specified	2
Treatment	
CROSS	18
MAGIC	8
Tumour Regression Grade	
1	5
2	5
3	9
4	6
5	1

Supplementary Table 3: Patient characteristics of patient cohort for *ex-vivo* study

	**n=6**
Male	5
Female	1
Median Age	68.5 (61-83)
Median BMI at diagnosis	30.15 (25.5-38.5)
Tumour Stage at diagnosis	
2	2
3	3
4	1
Nodal Status at diagnosis	
0	1
1	3
2	2
Treatment	
CROSS	3
MAGIC	1
Other Chemotherapy	2
Tumour Regression Grade	
3	1
4	1
5	1
Not Specified	3
